# Increasing X-ray energy improves data quality in serial crystallography

**DOI:** 10.1107/S1600577525011063

**Published:** 2026-01-21

**Authors:** Do-Heon Gu, Danny Axford, James Beilsten-Edmands, Sofia Jaho, Robin L. Owen

**Affiliations:** a Diamond Light Source Harwell Science and Innovation Campus DidcotOX11 0DE United Kingdom; University of Essex, United Kingdom

**Keywords:** high-energy X-rays, serial crystallography, radiation damage, serial synchrotron crystallography

## Abstract

Serial synchrotron crystallography (SSX) enables structure determination from microcrystals under near-physiological, room-temperature conditions but is limited in part due to the inevitable onset of radiation damage. The ability to reduce the absorbed dose while retaining, or even improving, data quality is an attractive means of mitigating this limitation. Advances in detector technology have made the use of high-energy X-rays a routine approach in MX, improving diffraction efficiency and enhancing overall data quality. Here, we systematically evaluate low-dose SSX data collected at five different X-ray energies from 12.4 to 25 keV using a CdTe Eiger2 detector while maintaining a constant dose. Higher photon energies increased the mean diffracted intensity and signal-to-noise ratio per unit dose, and facilitated higher-resolution structure determination, even with limited crystal numbers. These findings highlight the advantages of high-energy X-rays and provide practical guidance for optimizing SSX experiments in probing protein dynamics.

## Abstract 2

Advanced serial crystallography utilizing high-energy X-rays enhances diffraction efficiency, data quality and structural resolution under room-temperature conditions.

## 1. Introduction

Serial crystallography (SX) has emerged as a powerful technique for elucidating the dynamic behaviour of protein structures by enabling diffraction data collection from numerous microcrystals under near-physiological, room-temperature (RT) conditions (Schulz *et al.*, 2025 ▸; Henkel & Oberthür, 2024 ▸; Martin-Garcia, 2021 ▸; Fischer, 2021 ▸). In contrast to conventional macromolecular crystallography (MX), which usually employs cryo-cooled single crystals, SX typically utilizes a continuous stream or fixed-target support to deliver thousands of microcrystals into the X-ray beam, with each crystal contributing a single diffraction image. This approach minimizes radiation damage at RT and enables the capture of transient structural intermediates relevant to protein function.

Recent advances in X-ray sources, including synchrotron and XFEL facilities, as well as improvements in X-ray optics, have dramatically improved beam brightness and focusing capabilities, enabling high-quality data acquisition from extremely small crystals (Orville *et al.*, 2024 ▸). However, the concomitant increased beam intensity at the sample position increases the risk of radiation damage (Thorne, 2023 ▸; de La Mora *et al.*, 2020 ▸). This concern is especially critical in serial synchrotron crystallography (SSX), where, even though each crystal is exposed for only a single frame, crystals are typically held at RT. Therefore, optimization of experimental parameters to maximize data quality while minimizing the absorbed dose is a key aspect of experiment design (Shelley & Garman, 2022 ▸; Fischer, 2021 ▸).

Obtaining a complete and accurate dataset in SX typically requires the merging of thousands of diffraction images to achieve sufficient multiplicity and completeness. Under these conditions, maximizing diffraction efficiency per unit of absorbed dose is essential for minimizing radiation induced structural degradation. X-ray energy plays a pivotal role in determining this efficiency. Higher photon energies reduce photoelectric absorption within the crystal lattice, decreasing the deposited dose for a given incident flux (Shelley & Garman, 2024 ▸; Storm *et al.*, 2021 ▸; Fischer, 2021 ▸; Storm *et al.*, 2020 ▸). This reduction allows, in principle, for more diffraction patterns to be recorded before significant radiation damage occurs. Moreover, higher energy X-rays increase the elastic scattering cross section relative to absorption, enhancing the diffracted intensity per unit dose. The additional effect of photoelectron escape in microcrystals at elevated energies further reduces dose deposition, providing an intrinsic advantage for RT experiments (Marman *et al.*, 2018 ▸; Garman & Weik, 2017 ▸; Nave & Hill, 2005 ▸).

Despite these benefits, the practical application of high-energy X-rays in MX has historically been constrained by detector and X-ray source capabilities. The quantum efficiency of silicon based detectors reduces significantly above ∼15 keV, compromising their ability to detect high-energy diffraction signals, while the photon flux delivered by synchrotron and XFEL MX beamlines is often not optimized for energies above ∼20 keV.

The advent of hybrid photon counting detectors with cadmium telluride (CdTe) sensors has largely overcome the constraint imposed by silicon detectors. CdTe based detectors maintain high quantum efficiency (>90%) up to energies near the cadmium absorption edge (approximately 26.7 keV), with only modest reductions observed at even higher energies (Donath *et al.*, 2025 ▸; Collonge *et al.*, 2024 ▸; Zambon *et al.*, 2018 ▸). At the facility level, the emergence of fourth-generation synchrotron sources (*e.g.* APS, ESRF-EBS, MAX4^U^, and the upcoming Diamond-II) will dramatically improve brilliance and coherent flux at high energies (Ghasem *et al.*, 2024 ▸). Similarly, recent upgrades to XFEL facilities, including megahertz repetition rates and attosecond pulse generation, have significantly enhanced hard X-ray output (Yan *et al.*, 2024 ▸). Altogether, these technological advances provide a solid foundation for systematic high-energy SX experiments which enable both improved signal detection and sufficient photon flux for advanced data collection.

In this study, we systematically investigated the effect of X-ray energy on SSX data quality using a fixed-target setup with a CdTe Eiger2 detector. Datasets were collected at five photon energies (12.4, 17.5, 20, 23 and 25 keV) and key crystallographic metrics including mean intensity (〈*I*〉), signal to noise ratio [〈*I*/σ(*I*)〉], *R*_split_ and *CC*_1/2_ were evaluated under equivalent absorbed dose conditions. To minimize the impact of radiation damage, all experiments were carried out using extremely low absorbed doses of less than 0.5 kGy: some three orders of magnitude less than the suggested RT dose limit (de La Mora *et al.*, 2020 ▸). This approach minimizes radiation induced artefacts and facilitates a direct comparison of how X-ray energy influences diffraction performance. Through the comparison of datasets using five different incident beam energies, we aimed to quantify the advantages of high-energy X-rays and provide clear guidance for the optimization and design of SX experiments.

## 2. Methods

### 2.1. Sample preparation and sample mounting

Hen egg white lysozyme was purchased from Hampton Research (Cat No. HR7-110; Aliso Viejo, CA, USA). It was dissolved in 20 m*M* sodium acetate pH 4.5 at 100 mg ml^−1^ for crystallization. Crystals suitable for SSX were obtained via the batch method with seeding. The lysozyme solution (500 µl) was mixed with a crystallization buffer (buffer A) (500 µl) containing 20 m*M* sodium acetate pH 4.5, 1.2 *M* sodium chloride and 25%(*v*/*v*) ethyl­ene glycol. Crystals were grown for 12 h at RT and crushed to be less than 5 µm to act as seeds for batch crystallization. The batch crystallization for SX was conducted by mixing lysozyme (500 µl), buffer A (250 µl), 250 µl of buffer B [0.1 *M* sodium acetate pH 3.0, 6%(*w*/*v*) PEG 6000 and 20% sodium chloride] and the seed crystal slurry (1 µl). Crystals grew within 5 min at RT. The average dimensions of the grown crystals were approximately 12 µm × 10 µm × 8 µm at a concentration of 8 × 10^12^ crystals per ml.

The grown crystals were dispensed onto silicon chips with 11 µm-sized apertures, using a 40 µl aliquot of crystal slurry in a fully humidified, sealed chamber. The crystal suspension was evenly distributed across the chip surface and the excess buffer removed using a vacuum pump. Loaded chips were mounted on an aluminium holder and sealed with 6 µm Mylar film to prevent crystal dehydration as previously described (Horrell *et al.*, 2021 ▸) before transfer to the beamline for data collection.

### 2.2. Data collection

All SX data were collected at beamline I24, Diamond Light Source, using a fixed-target approach (Jaho *et al.*, 2024 ▸; Horrell *et al.*, 2021 ▸). SX datasets were collected at five X-ray energies (12.4, 17.5, 20, 23 and 25 keV). Prior to each data collection, the X-ray flux at each energy was measured using a PD300-500 silicon PIN diode (Canberra) as described by Owen *et al.* (2009 ▸). The X-ray beam was focused to 20 µm × 20 µm (FWHM) at the sample position. Based on the measured flux and resulting dose calculations using *RADDOSE-3D* (Zeldin *et al.*, 2013 ▸), the exposure time and beam attenuation were adjusted to achieve a diffraction-weighted dose of 0.43 kGy per frame for each X-ray energy. The incident (attenuated) photon flux and exposure times used at each energy were 1.0, 2.2, 2.9, 3.9 and 4.7 × 10^11^ photons s^−1^; and 10.5, 10.5, 9.2, 10 and 35.8 ms at 12.4, 17.5, 20, 23 and 25 keV, respectively. This low-dose approach was employed to minimize confounding factors such as radiation damage or dose-dependent intensity decay between energies, facilitating a reliable comparison of energy-dependent variations in diffraction quality.

Data collection was performed at RT (294 K) and data were recorded using a CdTe EIGER2 9M detector (Dectris, Switzerland). The sample-to-detector distance was adjusted for each X-ray energy to maintain a constant resolution of 1.4 Å at the detector edge (edge defined as inscribed circle). The respective sample-to-detector distances were 190, 285, 330, 385 and 420 mm for 12.4, 17.5, 20, 23 and 25 keV, respectively. All datasets were collected using identical sample preparation protocols and experimental conditions. Data were collected until a minimum of 12000 integrated lattices were obtained at each energy which typically required four chips. The average percentage of frames with multiple lattices recorded was 1.6% and this was consistent across both individual chips and energies, suggesting a near optimal loading protocol.

### 2.3. Data analysis

Diffraction data were processed using the *xia2.ssx* pipeline (Beilsten-Edmands *et al.*, 2024 ▸), which automates indexing, integration and scaling. Data reduction employed the *DIALS* (version 3.23) framework (Winter *et al.*, 2018 ▸), optimized for the high-throughput requirements of SX. Data frames with 20 or more strong spots found were subjected to an indexing attempt, and then integration was attempted where indexing was successful. During the indexing step, geometry optimization was performed iteratively to refine the beam centre position. Geometry refinement was repeated until the deviation in the *XY* plane from each diffraction image was minimized, ensuring accurate spot prediction and reliable indexing across individual frames. Indexing and integration were followed by scaling to place all measurements on an internally consistent scale and merging of observations to produce a final set of intensities. The indexing rate of hits at each energy was similar with no clear energy dependence observed.

To assess the effect of dataset size on data quality, we applied random sampling to the processed images. Subsets of data containing 5000, 7000, 8000, 9000, 10000 and 12000 integrated diffraction images were scaled and merged. Random sampling was repeated multiple times to minimize sampling bias, ensuring that the observed variations in diffraction quality represented intrinsic statistical effects rather than subset-specific anomalies. Subsets were combined using *dials.combine_experiments* and reprocessed using *xia2.ssx_reduce*. During the scaling step, the resolution cutoff was determined according to the criteria implemented in the *xia2.ssx_reduce* pipeline. In this procedure, no resolution cutoff is initially imposed, data are then scaled and merged, and the resolution cutoff determined as the point at which *CC*_1/2_ drops below 0.3. We found initial inclusion of all data beneficial for two main reasons. First, diffraction images were collected under extremely low-dose (0.43 kGy), single-hit conditions, resulting in individual reflections with inherently weak intensities. Secondly, unlike in conventional MX, reflections are typically observed only once per crystal, making the inclusion of marginal data essential. Subsequent statistical aggregation across thousands of crystals enables weak reflections to contribute meaningfully to electron density map quality and structural interpretation.

By employing these criteria, the energy dependence of data quality in SX could be more readily assessed without being obscured by overly stringent cutoffs. For all sub-datasets, the overall completeness was >99% and the multiplicity greater than 25-fold.

## 3. Results

The impact of increased X-ray energies on SX was evaluated through SSX experiments conducted using the fixed-target method at the I24 beamline of Diamond Light Source. Diffraction quality was systematically assessed using five key metrics [〈*I*〉, 〈*I*/σ(*I*)〉, *R*_split_, *CC*_1/2_ and the resolution limit], across five different X-ray energies and varying subset sizes from 5000 to 12000 crystals.

**Figure 1:**
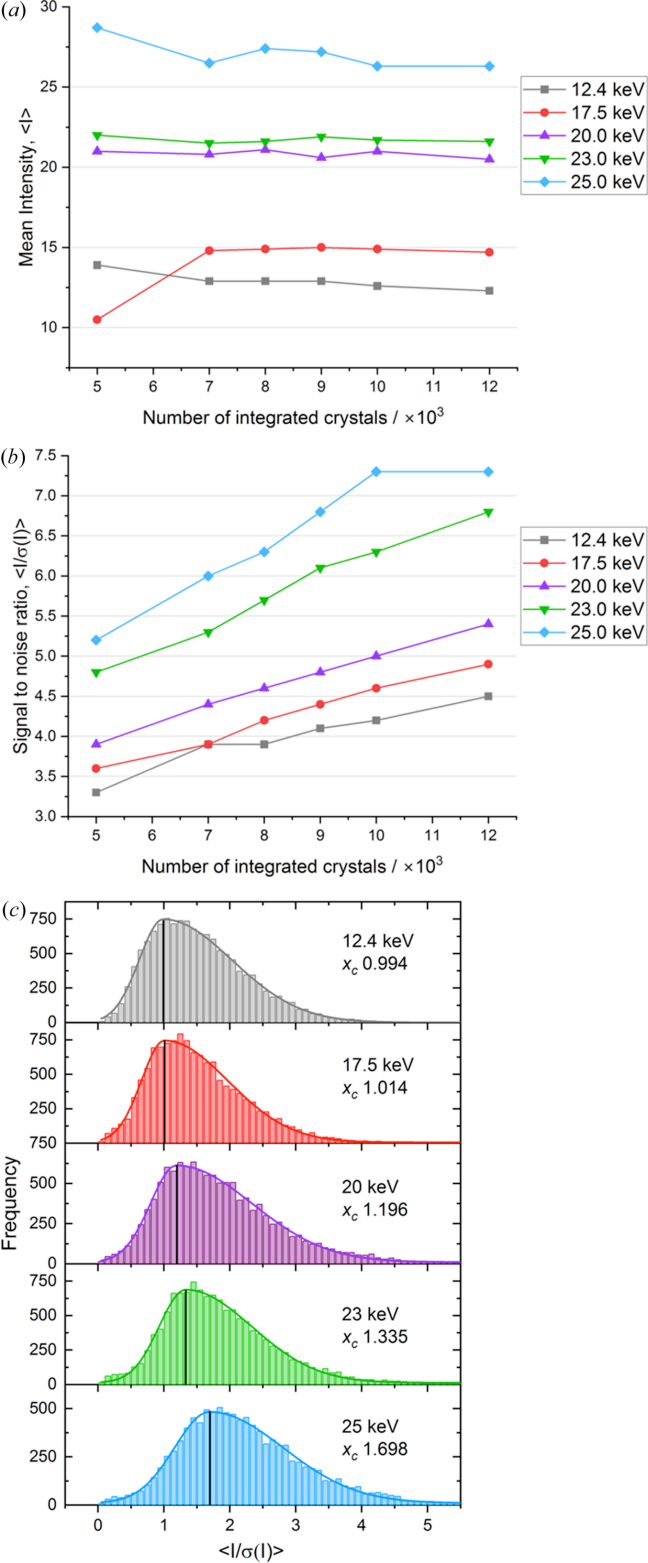
Energy-dependent changes in mean diffraction intensity and signal-to-noise ratio as dataset size increases. The grey, red, purple, green and blue lines correspond to data collected at 12.4, 17.5, 20, 23 and 25 keV, respectively. (*a*) Mean diffraction intensities (〈*I*〉) plotted against the number of integrated images from 5000 to 12000. The corresponding multiplicity for each dataset ranged from 26-fold (5k crystals) to 57-fold (12k crystals) for the 25 keV data. (*b*) Mean dataset signal-to-noise ratio, 〈*I*/σ(*I*)〉, plotted against the number of integrated images. (*c*) Mean signal-to-noise ratio, 〈*I*/σ(*I*)〉, for individual crystals at each energy (all data).

The mean diffraction intensity (〈*I*〉) for each X-ray energy as a function of the number of integrated images is shown in Fig. 1 ▸(*a*). For a constant absorbed dose, the 25 keV dataset consistently occupying the uppermost 〈*I*〉 position, followed by 23, 20, 17.5 and 12.4 keV in descending order. Quantitatively, when normalized across all dataset sizes, mean 〈*I*〉 increased by 9.4, 61.3, 68.1 and 109.5% for 17.5, 20, 23 and 25 keV data, respectively, relative to 12.4 keV.

Reflecting the trend observed in the mean diffraction intensity, the mean signal-to-noise ratio [〈*I*/σ(*I*)〉] increases as a function of energy, but also of dataset size [Fig. 1 ▸(*b*)]. The improvement in 〈*I*/σ(*I*)〉 with dataset size is primarily driven by statistical averaging, which reduces random error and enhances measurement precision, even though the absolute diffracted intensity remains approximately constant. However, the upward trend in 〈*I*/σ(*I*)〉 with increasing photon energy reflects a more fundamental advantage that goes beyond statistical effects with high-energy datasets consistently being superior. Even at 5000 images, the 25 keV data exhibited 〈*I*/σ(*I*)〉 values comparable to, or exceeding, those of larger lower-energy datasets. This indicates that higher photon energies not only boost absolute intensity but also reduce relative noise, yielding more accurate measurements at equivalent absorbed dose. When normalized across the full resolution range, 〈*I*/σ(*I*)〉 improved by 7.1, 17.5, 46.2 and 62.5% for 17.5, 20, 23, and 25 keV, respectively, compared with 12.4 keV. These results were validated by plotting histograms showing the distribution of 〈*I*/σ(*I*)〉 at the individual crystal level, across the different photon energies [Fig. 1 ▸(*c*)]. The consistent shape of the distributions but with a shift to higher values of 〈*I*/σ(*I*)〉 with increasing energy indicates that the observed improvements in 〈*I*/σ(*I*)〉 are not due to bias arising from preferential selections during scaling and downstream processing, but rather reflect the intrinsic advantage high-energy X-rays provide.

These visual and quantitative trends demonstrate that increasing X-ray energy systematically enhances diffraction efficiency per unit dose, yielding both higher mean 〈*I*〉 and improved 〈*I*/σ(*I*)〉 across all dataset sizes. The consistency of these gains suggests that high-energy data collection could be especially advantageous for crystal-limited SX experiments. These results are also consistent with theoretical expectations and previous reports (Storm *et al.*, 2021 ▸), which proposed that higher X-ray energies provide greater diffraction efficiency per unit dose under cryogenic conditions.

**Figure 2:**
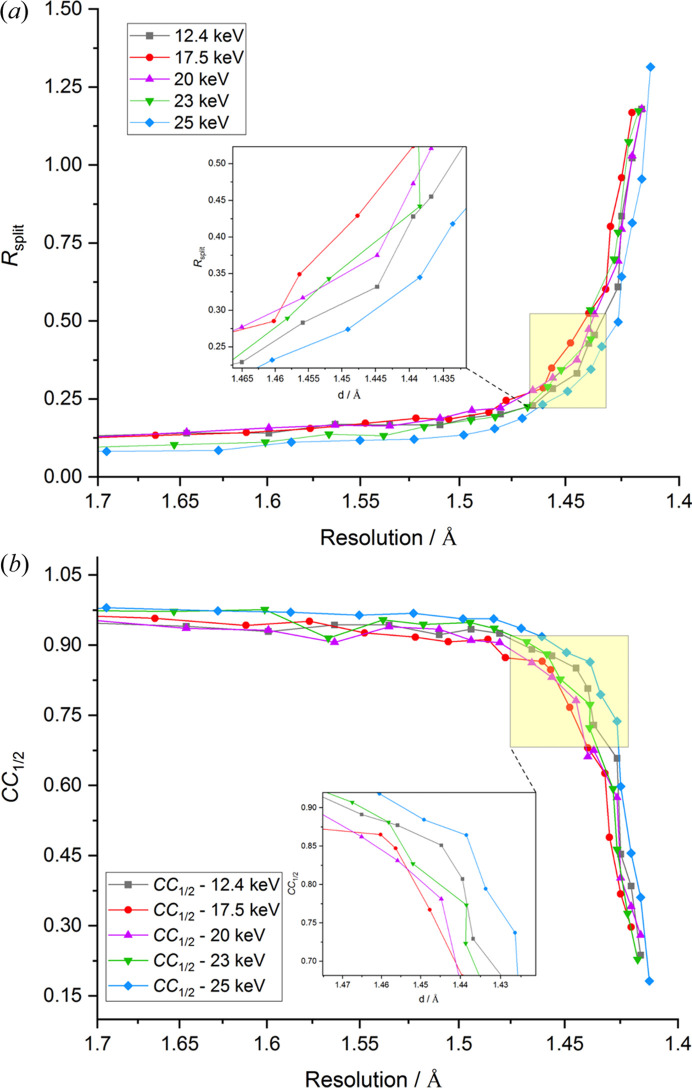
Energy-dependent changes in (*a*) *R*_split_ and (*b*) *CC*_1/2_ based on datasets comprising 12000 integrated images. The grey, red, blue, green and purple lines represent data collected at 12.4, 17.5, 20, 23 and 25 keV, respectively. The plot regions highlighted in yellow are enlarged in the insets. Wilson plots of these datasets are shown in Fig. S1 of the supporting information.

The energy-dependent improvements observed in mean 〈*I*〉 and 〈*I*/σ(*I*)〉 were also reflected in metrics evaluating data consistency and reliability. As shown in Figs. 2 ▸(*a*) and 2 ▸(*b*), *CC*_1/2_ values generally increased and *R*_split_ values decreased as a function of X-ray energies. The 12.4 keV dataset represents an exception to this trend, exhibiting slightly higher *CC*_1/2_ and lower *R*_split_ values compared with 23 keV in certain subsets. Despite this anomaly, other datasets (17.5–25 keV) vary consistently demonstrating improved *R*_split_ and *CC*_1/2_ as a function of energy, reflecting more efficient diffraction per unit dose. For a given absorbed dose, high-energy X-rays generate stronger diffraction and enable high-quality data acquisition with fewer crystals, underscoring their overall advantage for SX experiments.

**Figure 3:**
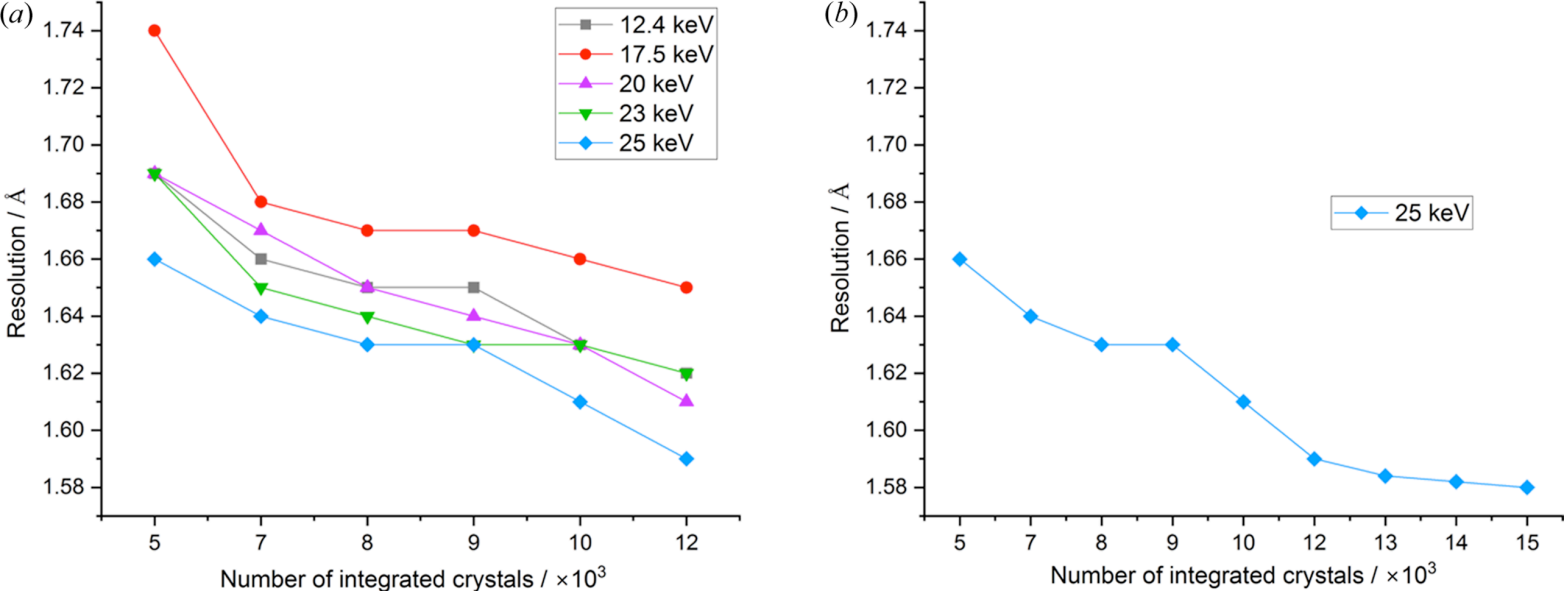
High resolution cutoff as a function of energy and dataset size. (*a*) Maximum resolution obtained from datasets collected at 12.4, 17.5, 20, 23 and 25 keV. The black, red, blue, green and purple lines represent the respective energies. (*b*) Maximum resolution of datasets collected at 25 keV, compared across an extended range of integrated images.

To assess the impact of photon energy on the resolution limit, the high-resolution cutoff of all sub-datasets were determined at *CC*_1/2_ ≥ 0.3, as described in Section 2.3. The advantage of high-energy X-rays was again evident, with most comparisons showing an improved trend in resolution at higher energies [Fig. 3 ▸(*a*)]. We note that the resolution at the edge of the detector (1.4 Å) was beyond that obtained from the highest-resolution datasets. The 25 keV dataset showed a consistent resolution gain of 0.04–0.09 Å compared with 17.5 keV, across subsets from 5000 to 12000 images. The resolution gains, while consistent, are less pronounced than might be expected from Fig. 1 ▸(*b*), and we postulate that this reflects the unsuitability of using *I*/σ(*I*) to define the resolution of serial datasets. These findings suggest that, although lower-energy datasets occasionally show better resolution than their high-energy counterparts, in general high-energy data collection yields higher resolution data, even when the number of microcrystals is limited.

This raises the question of how much further data resolution and data scaling improve as a function of dataset size. To address this, additional 25 keV data were processed using up to 15000 indexed lattices. The resolution limit steadily improves with increasing dataset size up to approximately 13000 crystals but plateaued at 1.58 Å beyond this [Fig. 3 ▸(*b*)]. This observation suggests that beyond a certain dataset size, additional data accumulation provides diminishing returns in resolution improvement.

**Figure 4:**
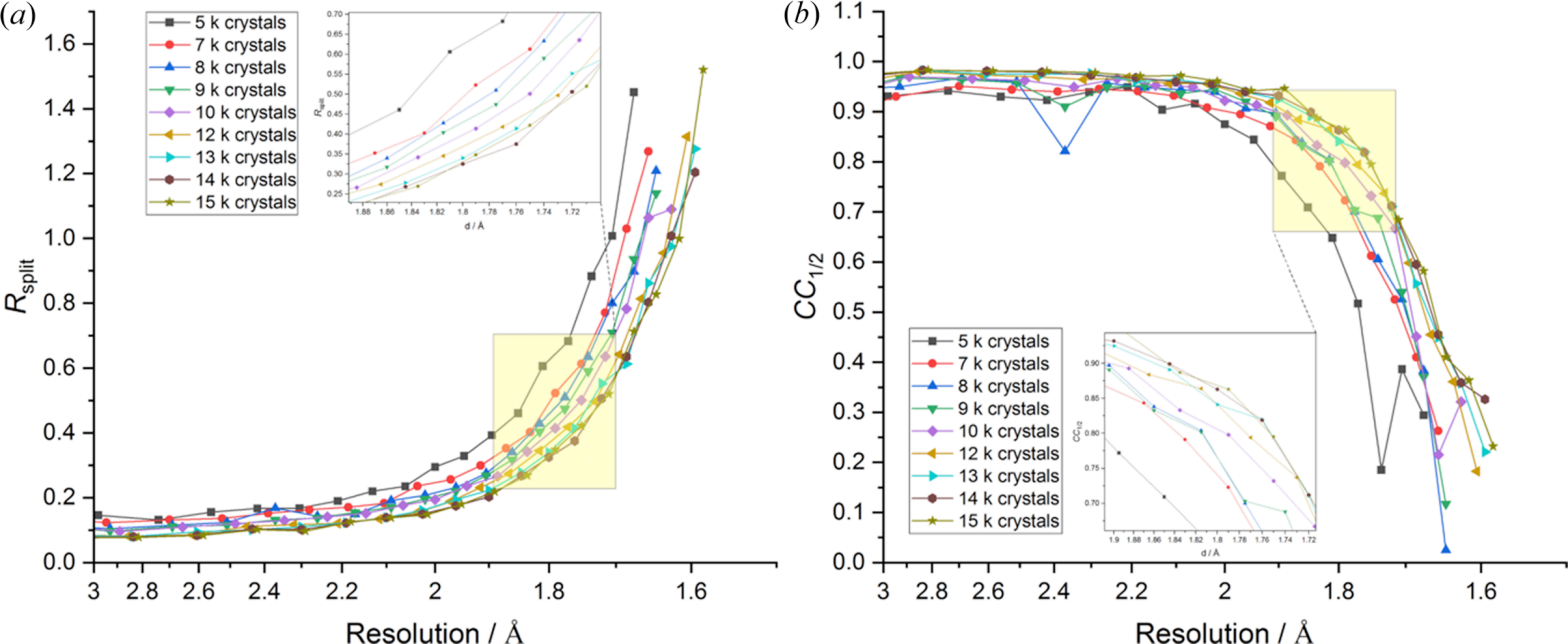
*R*_split_ and *CC*_1/2_ changes for the 25 keV datasets with different numbers of integrated images as a function of resolution. Inflection points highlighted in yellow are enlarged in the insets. (*a*) *R*_split_ and (*b*) *CC*_1/2_ plotted against resolution for each dataset.

The data quality indicators *R*_split_ [Fig. 4 ▸(*a*)] and *CC*_1/2_ [Fig. 4 ▸(*b*)] show a similar trend as a function of dataset size. Comparison of datasets collected at 25 keV, encompassing 5000 to 15000 crystals, show that increasing dataset size results in a progressive reduction in *R*_split_ values accompanied by a concomitant increase in *CC*_1/2_, with the most pronounced improvements observed in higher-resolution shells. The decrease in *R*_split_ is substantial; relative to the 5000 crystal dataset (*R*_split_ = 0.205), the 7000, 8000, 9000, 10000, 12000, 13000, 14000 and 15000 crystal datasets exhibited progressive reductions in *R*_split_ of 4.59, 17.82, 24.24, 26.54, 50.74, 62.70, 65.32 and 65.32%, respectively. The corresponding multiplicities of each dataset was 27.1, 35.7, 39.9, 45.04, 48.9, 57.1, 61.0, 65.8 and 68.2. As expected, these data clearly demonstrate that larger datasets substantially enhance data accuracy. However, the rate of improvement in *R*_split_ begins to reach a plateau beyond 13000 crystals, suggesting diminishing returns despite the continued increase in multiplicity.

This trend of diminishing returns is consistently reflected in both Figs. 3 ▸(*b*) and 4 ▸. Initial increases in dataset size lead to substantial improvements in resolution [Fig. 3 ▸(*b*)] and data quality metrics such as *R*_split_ and *CC*_1/2_ (Fig. 4 ▸). As the number of integrated images continues to grow, the rate of improvement reduces. These observations demonstrate that, although larger datasets contribute positively to resolution, accuracy and internal consistency, the extent of such improvements is ultimately constrained by the intrinsic diffraction properties of the crystals. Additionally, improvement of dataset quality to a given threshold is reached using a lower number of indexed/integrated images as the X-ray energy increases. The observed plateau in internal consistency beyond a certain dataset size may however also reflect technical limitations in current data processing algorithms for SX, which generally exhibit lower accuracy compared with those used for rotation data. Therefore, in addition to crystal properties, improvements in data processing algorithms represent a critical factor for enhancing the interpretation and overall quality of SX data.

To assess if inadvertent selection bias, *i.e.* preferentially selecting stronger diffraction images for analysis, was a reason for observing improved data quality as a function of energy, indexing and integration rates were assessed. However, no correlation between the X-ray energy and indexing or integration rate was observed, with chip-to-chip variation greater than any energy dependence, supporting the premise that enhancements in data quality are attributable to the advantages of high-energy X-rays.

## 4. Discussion

This study demonstrates that in SX, increasing the X-ray photon energy under equivalent absorbed dose conditions leads to systematic improvements in diffraction efficiency and data quality. SX datasets collected at higher photon energies, ranging from 17.5 to 25 keV, showed increased mean diffraction intensity, improved scaling statistics and higher resolution cutoff compared with those collected at the more commonly used energy of 12.4 keV (λ ≃ 1 Å).

The trend of improving data quality metrics with increasing energy can be seen to be imperfect, with some subsets of lower-energy-acquired measurements looking better than some subsets acquired at higher energies. This is perhaps expected given the nature of the SSX experiment, with its sparse sampling of reflection intensities with monochromatic beam, implying elevated measurement uncertainties and crystal-to-crystal variation. However, the overall picture leaves little ambiguity as to the presence of a beneficial effect with increasing energy.

High-energy datasets further demonstrated clear improvements in achievable resolution, particularly at the detector edges. However, analysis of the 25 keV dataset up to 15000 crystals showed that resolution plateaued near 1.58 Å beyond 13000 crystals, indicating that additional data accumulation yields diminishing returns in resolution. This suggests that, beyond a certain dataset size, intrinsic factors such as crystal quality and lattice order become the primary determinants of attainable resolution.

In contrast, measures of data accuracy and internal consistency, including *R*_split_ and *CC*_1/2_, continued to improve systematically with increasing dataset size. Across datasets from 5000 to 13000 crystals, *R*_split_ progressively decreased while *CC*_1/2_ increased, with the most pronounced improvements observed in higher-resolution shells. These results indicate that while larger datasets substantially enhance reliability and internal consistency, the rate of improvement diminishes beyond 13000 crystals despite continued increases in multiplicity. This suggests that, although accuracy metrics benefit from data accumulation, the practical gains become progressively smaller once a sufficiently large dataset has been collected. Especially under conditions of limited crystal availability, a higher incident photon energy facilitates collection of optimal diffraction data per unit absorbed dose.

Overall, these findings underscore the benefits of using high-energy data collection in SX. Optimization of beamline design and experimental parameters can maximize and significantly improve data quality, and this is especially critical when limited crystals are available. High-energy serial data collection, facilitated by both suitable detectors and emerging X-ray sources capable of delivering higher photon energies, enables stronger diffraction signals, improved signal-to-noise ratios and higher-resolution structural information to be obtained from a given set of crystals and a given, or tolerable, absorbed dose. These results align closely with theoretical predictions and prior studies (Storm *et al.*, 2021 ▸), which suggest that higher X-ray energies improve diffraction efficiency per unit dose, regardless of the temperature conditions.

This study underscores the broad applicability and advantages of employing higher X-ray energies in both MX and SX, while also providing guidance for future beamline developments. Importantly, these benefits are effectively ‘cost-free’ from a user perspective, as they do not necessitate additional instrumentation or increased experimental complexity. Although demonstrated here in the context of SSX, the energy dependence of *I*/σ(*I*), *R*_split_ and *CC*_1/2_ are expected to confer comparable advantages in serial femtosecond crystallography despite the less pressing need to minimize dose-per-frame. With the advent of next-generation synchrotron and XFEL sources capable of delivering enhanced flux at higher energies, further improvements in data quality and experimental efficiency can be anticipated.

## Supplementary Material

Supporting Figure S1. DOI: 10.1107/S1600577525011063/rv5200sup1.pdf

## Data Availability

Raw data are available upon request.
