# Supplementary material for: Increasing X-ray energy improves data quality in serial crystallography
Source: J Synchrotron Radiat. 2026 Jan 21;33(Pt 2):344–50. doi: 10.1107/S1600577525011063 (PMC12948025; doi:10.1107/S1600577525011063)
Supplement: Supplementary file 1 [file s-33-00344-sup1.pdf]

JOURNAL OF SYNCHROTRON RADIATION

Volume 33 (2026)

Supporting information for article:

Increasing X-ray energy improves data quality in serial crystallography

Do-Heon Gu, Danny Axford, James Beilsten-Edmands, Sofia Jaho and Robin

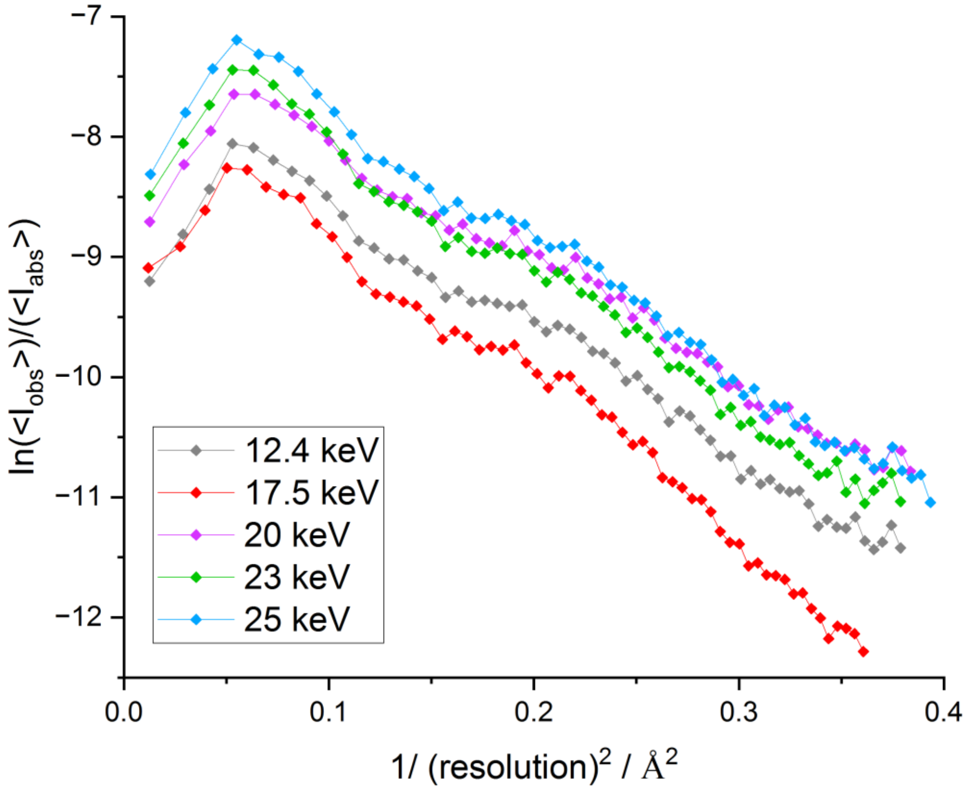

Figure S1 Wilson plots for the data collected at 12.4, 17.5, 20, 23 and 25keV.
